# Intravenous AAV9 efficiently transduces myenteric neurons in neonate and juvenile mice

**DOI:** 10.3389/fnmol.2014.00081

**Published:** 2014-10-15

**Authors:** Sara E. Gombash, Christopher J. Cowley, Julie A. Fitzgerald, Jodie C. E. Hall, Christian Mueller, Fedias L. Christofi, Kevin D. Foust

**Affiliations:** ^1^Department of Neuroscience, Ohio State UniversityColumbus, OH, USA; ^2^Department of Neuroscience, Center for Brain and Spinal Cord Repair, Ohio State UniversityColumbus, OH, USA; ^3^Department of Pediatrics, Gene Therapy Center, University of Massachusetts Medical SchoolWorcester, MA, USA; ^4^Department of Anesthesiology, Ohio State UniversityColumbus, OH, USA

**Keywords:** AAV9, adeno-associated virus, enteric nervous system, myenteric plexus, functional gastrointestinal motility disorders, enteric glia, enteric neuropathy, gene therapy

## Abstract

Gene therapies for neurological diseases with autonomic or gastrointestinal involvement may require global gene expression. Gastrointestinal complications are often associated with Parkinson's disease and autism. Lewy bodies, a pathological hallmark of Parkinson's brains, are routinely identified in the neurons of the enteric nervous system (ENS) following colon biopsies from patients. The ENS is the intrinsic nervous system of the gut, and is responsible for coordinating the secretory and motor functions of the gastrointestinal tract. ENS dysfunction can cause severe patient discomfort, malnourishment, or even death as in intestinal pseudo-obstruction (Ogilvie syndrome). Importantly, ENS transduction following systemic vector administration has not been thoroughly evaluated. Here we show that systemic injection of AAV9 into neonate or juvenile mice results in transduction of 25–57% of ENS myenteric neurons. Transgene expression was prominent in choline acetyltransferase positive cells, but not within vasoactive intestinal peptide or neuronal nitric oxide synthase cells, suggesting a bias for cells involved in excitatory signaling. AAV9 transduction in enteric glia is very low compared to CNS astrocytes. Enteric glial transduction was enhanced by using a glial specific promoter. Furthermore, we show that AAV8 results in comparable transduction in neonatal mice to AAV9 though AAV1, 5, and 6 are less efficient. These data demonstrate that systemic AAV9 has high affinity for peripheral neural tissue and is useful for future therapeutic development and basic studies of the ENS.

## Introduction

The recent identification of adeno-associated viral vector (AAV) serotypes that cross the blood brain barrier (BBB) resulted in unprecedented therapeutic benefit in animal models of neurological disease (Foust et al., 2010, 2013; Fu et al., 2011; Ahmed et al., 2013; Garg et al., 2013; Haurigot et al., 2013). AAV9 was the first serotype described that efficiently crossed the BBB (Foust et al., 2009). Subsequently multiple groups have demonstrated that intravenous injection of AAV9 into mice, rats, cats and non-human primates produces robust transduction in the central nervous system (CNS) (Duque et al., 2009; Tatom et al., 2009; Bevan et al., 2011). Intravenous injection of AAV9 in neonatal mice results in primarily neuronal transduction throughout the brain and spinal cord, but preferentially targets astrocytes when injected in mice greater than 2 weeks of age (Foust et al., 2009). Similar CNS transduction patterns have been reported with other AAV serotypes following systemic delivery (Zhang et al., 2011). AAV's affinity for nervous tissue suggests that the peripheral nervous systems are likely transduced following systemic injection. Gene delivery to large peripheral neuronal networks such as the autonomic nervous system (ANS) and enteric nervous system (ENS) may be beneficial for treatment of global neurological disease. To date, transduction in peripheral neurons has not been thoroughly investigated.

The ENS is often referred to as “the little brain in the gut” and is estimated to have as many neurons as the spinal cord (Wood, 2000). The ENS is embedded within the mucosal and muscular layers throughout the length of the gastrointestinal (GI) tract and enteric neurons and glial cells are arranged into two ganglionated plexuses called the submucosal and myenteric plexus, respectively. The submucosal plexus senses and reacts to stretch and chemical changes induced by luminal contents while the myenteric plexus coordinates motor function of smooth muscle. Dysfunction of the ENS is linked to a series of GI disorders that are often chronic and prevalent in pediatric and adult populations (Fukudo et al., 2012; Yeung and Di Lorenzo, 2012). For example, functional GI motility disorders (FGIMD) are estimated to affect 5–30% of adults and children in the United States (Saito et al., 2002; Camilleri et al., 2005). In FGIMDs, disruption of GI nerves and/or muscle can cause chronic gastroesophageal acid reflux, constipation, abdominal pain and bloating. However, etiology is not well understood and suitable long-term therapies do not exist (Fukudo et al., 2012). The ENS is also implicated in functional gastrointestinal disorders (FGIDs) such as dyspepsia and irritable bowel syndrome that affect 15–20% of the US population. Further, the ENS is a potential therapeutic target for diarrheal disorders and inflammatory bowel diseases (IBD). GI neuromuscular disorders, such as chronic intestinal pseudo-obstruction (CIP), are also poorly understood (Gabbard and Lacy, 2013). In some CIP cases, myenteric neurons contain proteinaceous nuclear inclusions and evidence of apoptosis, presumably causing the GI dysfunction (El-Rifai et al., 2006; Gabbard and Lacy, 2013). Interestingly, GI dysfunction also commonly occurs in patients with autism or Parkinson's disease (Anderson et al., 2007; Buie et al., 2010; Wakabayashi et al., 2010; Natale et al., 2011; Mazurek et al., 2012). Parkinson's disease Lewy bodies in ENS neurons can be identified in tissue biopsies (Derkinderen et al., 2011), suggestive that peripheral neurons are also affected in CNS diseases. Beyond diseases, aging is a risk factor for GI dysfunction (Camilleri et al., 2008; Wiskur and Greenwood-Van Meerveld, 2010). Chronic constipation is common in the elderly and can be associated with idiopathic ENS neuron degeneration (Sanchez and Bercik, 2011).

In addition to neurons, the ENS contains enteric glial cells (EGCs) that are similar to CNS astrocytes. Enteric glia, like CNS astrocytes, are essential for maintaining homeostasis and regulating neural circuit activity (Gulbransen and Sharkey, 2012). EGCs likely serve as key regulators of intestinal inflammation in animals and humans. Their ability to mediate immune responses *in vivo* was suggested as a possible pathologic mechanism in Crohn's Disease (Cornet et al., 2001). Data suggest that there is an impairment of the glial network in non-inflamed regions of the gut mucosa in patients with Crohn's Disease, as evidence by a decrease in GFAP immunoreactivity in glia (Cornet et al., 2001). Overall, EGCs like astrocytes in the brain mediate glial transmission, and regulate synaptic signaling, synaptic plasticity, network excitability and inflammation. EGCs contribute to the onset and development of intestinal inflammation' and are important in the understanding of GI inflammation occurring in IBD, enterocolitis, and gut infections (Savidge et al., 2007; Vijayaraghavan, 2009; Cirillo et al., 2011; McClain et al., 2014; Turco et al., 2014).

Together, the lack of available therapies for ENS is a major health problem and is an urgent need. Due to its safety and sustained expression, systemic AAV gene therapy may be a useful approach to treat and study the ENS and its associated disorders. AAV transduction of the ENS has been reported but not well characterized (Fu et al., 2011; Rahim et al., 2011; Mattar et al., 2013; Schuster et al., 2014) likely due to the unique architecture and intricate dissection techniques required for study. The goal of the current work was to characterize AAV9 transduction efficiency and cell types targeted in the myenteric plexus following intravenous injection into neonatal or juvenile mice. In contrast to age dependent transduction patterns in the mouse CNS (Foust et al., 2009), we show that self complementary AAV9 injection results in extensive myenteric neuron transduction at both neonate and juvenile time points in all regions of the GI tract. Furthermore, AAV9 transduction of EGCs pales in comparison to CNS astrocytes. Additionally, we also examined transduction of self complementary AAV serotypes 1, 5, 6, and 8 in the myenteric plexus and show that they differ greatly in transduction efficiency.

## Materials and methods

### Animals

A total of 20 male or female FVB mice were used for these studies. Postnatal day 1 (P1) pups were used in all neonatal injection studies and juvenile mice were used beginning at postnatal day 21 (P21). Following vector injection procedures, neonatal mice remained with the dam until weaning. Mice were housed with same-sex littermates and given food and water *ad libitum* in a constant 12 h light/dark cycle room in the AAALAC approved Ohio State University Biomedical Research Tower vivarium. All animal procedures were approved by the Ohio State University Institutional Laboratory Animal Care and Use Committee.

### AAV vector production and purification

All vectors used in these studies were produced by the University of Massachusetts Medical School Viral Vector Core. Self complementary AAV (scAAV) genomes were engineered to encode the green fluorescent protein (GFP) transgene under the control of the chicken-β-actin/cytomegalovirus hybrid (CB) promoter, flanked by AAV2 inverted terminal repeats. Virus was packaged by triple transfection of HEK293 cells with an adenovirus helper plasmid and plasmids containing the AAV2 *rep* gene and AAV1, 5, 6, 8, or 9 *cap* genes. Vectors were purified by cesium chloride gradient and titers were determined by qPCR. Vector titers were scAAV1- 2.4 × 10^13^, scAAV5- 9.0 × 10^12^, scAAV6- 5.0 × 10^12^, scAAV8- 1.0 × 10^13^, and scAAV9- 1.0 × 10^13^ vector genomes (vg)/ml.

### AAV injections

Intravaenous injections of scAAV9-CB-GFP or scAAV8-CB-GFP in neonatal (P1) and juvenile (P21) mice were completed as previously described (Foust et al., 2009; Gombash Lampe et al., in press)

### Neonates

A dissecting microscope and fiber-optic light source were used for visualization of the temporal face vein. First, neonatal mice were rested on a bed of ice for anesthetization and a 3/10 cc 30 gauge insulin syringe (Terumo Medical Corp., Elkton, MD) was loaded with 1 × 10^11^ vg/ml scAAV9-CB-GFP or 5 × 10^10^ vg/ml scAAV1, 5, 6, 8, or 9-CB-GFP. Viral particles supplemented with phosphate buffered saline (PBS, 0.01 M, pH 7.4) for a total volume of 50 μl were injected into the face vein. Pups were then warmed, returned to their home cage, and rubbed in bedding to prevent rejection by the mother. Neonatal animals were sacrificed 30–60 days post-injection for myenteric analysis.

### Juveniles

Juvenile tail vein injections were performed on P21 mice. Mice were restrained in an illuminated tail vein injection platform (Braintree Scientific Inc., Braintree, MA). Prior to injection, the tail was swabbed with alcohol, then 3/10 cc 30 gauge insulin syringes were used to inject scAAV9-CB-GFP for a final dose of 2 × 10^12^ vg. Mice were then returned to their home cages.

### Whole mount preparations

Whole mount longitudinal muscle-myenteric plexus (LMMP) tissues were microdissected as previously described (Wang et al., 2007). Briefly, the entire gastrointestinal tract beginning at the lower esophagus through the rectum was removed from euthanized mice and rinsed in room temperature PBS. Next, regions of the gastrointestinal tract (stomach, duodenum, jejunum, ileum, cecum, and colon) were separated, opened, and pinned flat with the luminal side up on a Sylgard (Dow Corning, Midland, MI) base in 100 mm cell culture dishes. Under a dissecting microscope, fine forceps were used to remove the muscosa, submucosa, and circular muscle to expose the myenteric plexus on the surface of the longitudinal muscle. Immunohistochemistry was then used to observe transgene expression, neurons, and glial cells within the myenteric plexus.

### Immunohistochemistry

LMMP tissues were fixed in Zamboni's fixative (4% paraformaldehyde and 0.2% piciric acid in 0.1 M PBS; #1459, Newcomer Supply, Middleton, WI) overnight at 4°C. The next day, tissues were rinsed in PBS until clear. Tissues were transferred to glass slides and incubated in blocking buffer [10% normal donkey serum (NDS), 0.5% Triton-X 100 in PBS] for 2 h at room temperature. Tissues were then incubated in appropriate primary antisera overnight at 4°C against green fluorescent protein (GFP; 1:500; ab13970, Abcam, Cambridge, MA), HuD (1:25; A-21271, Life Technologies, Eugene, OR), S100 Protein Ab-2 (S100; 1:200; RB-044-A0, Thermo Scientific, UK), choline acetyltransferase (ChAT; 1:50; AB144P, Millipore, Temecula, CA); vasoactive intestinal peptide (VIP; 1:200; Abcam), neuronal nitric oxide synthase (nNOS; 1:200; ab1376, Abcam), calretinin (1:200; CG1, Swant, Switzerland), or calbindin D-28K (1:200; AB1778, Millipore). The next day, tissues were rinsed 4 times for 10 min in PBS, then incubated in appropriate Alexa Fluor secondary antibodies at 1:200 for 2 h at room temperature. Antibody dilutent was 3% NDS, 0.5% Triton-X 100 in PBS. Tissues were then rinsed and slides were coverslipped with 2.5% PVA/DABCO. Fluorescent images were captured on an Olympus FV 100 Spectral Confocal system (Melville, NY) in the Ohio State University Campus Microscopy and Imaging Facility.

### Quantification of myenteric neurons

Fluorescently stained whole mount tissues were viewed with a Zeiss fluorescent microscope equipped with the appropriate filters to distinguish CY3, CY5, and FITC flurophores. Myenteric ganglia in the stomach, duodenum, jejunum, ileum, cecum, and colon were viewed at 40× for counting of HuD positive and GFP positive cells. Total neurons counted in each region are recorded in **Figure 3A**. HuD and GFP positive neuron counts were collected only from the colon for scAAV8-CB-GFP and low-dose scAAV9-CB-GFP (5 × 10^10^ vg) injected mice. For oral-aboral preference analysis in the colon, LMMP colon preparations in the correct orientation were separated into nine 1 cm segments. Numbers of HuD and GFP positive neurons in 10 ganglia in each segment were recorded, resulting in approximately 5000 HuD positive neurons counted within groups. Data is reported as the percentage of GFP positive neurons ± standard error of the mean (SEM).

### Statistics

A one-way repeated measures analysis of variance was used to compare percentages of neuronal transduction across the oral to aboral axis of the colon in neonatal and juvenile injected mice. A Student's *t*-test was used to compare transduced neuron counts following scAAV8-CB-GFP or scAAV9-CB-GFP injection. All results are listed as the mean ± s.e.m.

## Results

### AAV9 efficiently transduces myenteric neurons following intravenous injection into neonate and juvenile mice

**Figure 1:**
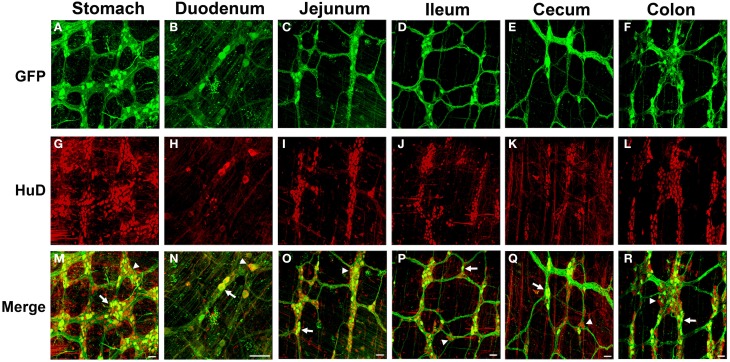
**Green fluorescent protein (GFP) expression in the myenteric plexus following intravenous injection of scAAV9-CB-GFP into neonatal mice**. Intravenous injection of scAAV9-CB-GFP into P1 mice resulted in GFP (green, **A–F**) expression in neurons (HuD, red, **G–L**) in the stomach **(A,G,M)**, duodenum **(B,H,N)**, jejunum **(C,I,O)**, ileum **(D,J,P)**, cecum **(E,K,Q)**, and in the colon **(F,L,R)**. Arrows in merged images **(M–R)** point to GFP expressing neurons. Arrowheads identify untransduced neurons. Scale bars are 100 μm.

**Figure 2:**
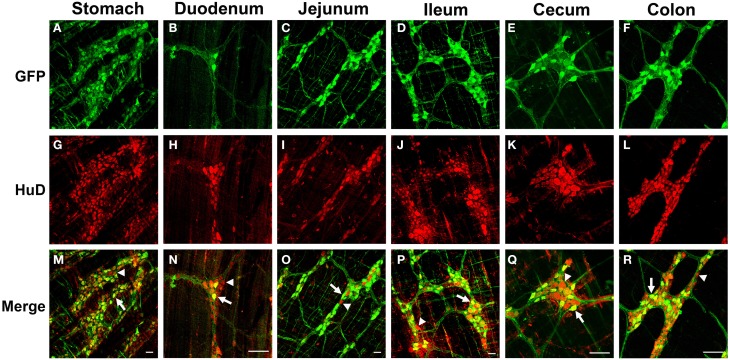
**Green fluorescent protein (GFP) expression in the myenteric plexus following intravenous injection of scAAV9-CB-GFP into juvenile mice**. Intravenous injection of scAAV9-CB-GFP into P21 mice resulted in robust GFP (green, **A–F**) expression in neurons (HuD, red, **G–L**) in the stomach **(A,G,M)**, duodenum **(B,H,N)**, jejunum **(C,I,O)**, ileum **(D,J,P)**, cecum **(E,K,Q)**, and in the colon **(F,L,R)**. Arrows in merged images **(M–R)** point to GFP expressing neurons. Arrowheads identify untransduced neurons. Scale bars are 100 μm.

To investigate AAV9 ENS transduction, neonatal mice (P1) were injected in the temporal vein with 1 × 10^11^ vg of self complementary (sc) AAV9-CB-GFP. Three weeks post-injection, immunolabeling for GFP of the myenteric plexus revealed robust, widespread expression in neuronal cell bodies and fibers. GFP expression was detected in all regions of the gastrointestinal tract, including the esophagus (not shown), stomach, duodenum, jejunum, ileum, cecum, and the colon (Figure 1). GFP localized in HuD positive cells in all regions of the GI tract. No GFP expression was observed in S100 positive enteric glia in any region, suggesting expression was confined to myenteric neurons (Supplementary Figure 1). Intravenous AAV9 preferentially targets neurons in neonatal mice and astrocytes in juvenile injected animals (Foust et al., 2009). To determine if ENS transduction also followed this pattern, 2 × 10^12^ vg of scAAV9-CB-GFP was injected into the tail vein of P21 mice. Similarly to injected neonates, GFP and HuD immunolabeling revealed widespread, robust transgene expression through all regions of the gastrointestinal tract (Figure 2). GFP expression was again limited to myenteric neurons and their projections and was not expressed in intraganglionic glial cells along the entire tract. The paucity of enteric glia transduction was surprising because we previously reported that scAAV-CB-GFP expression was highly efficient in CNS astrocytes (Foust et al., 2009). Others have shown that the neuronal tropism of AAV vectors can be redirected to glia by driving transgene expression under the GFAP promoter (Burger et al., 2004; Taymans et al., 2007; Lawlor et al., 2009; von Jonquieres et al., 2013; de Leeuw et al., 2014). GFAP expression is common to CNS astrocytes and enteric glia (Gulbransen and Sharkey, 2012). Therefore, we engineered a single stranded AAV9-GFAP-GFP vector and intravenously injected neonate mice with 1 × 10^11^ vg (*n* = 3). Mice were euthanized between 30 and 60 days post-injection and the myenteric plexus was labeled for GFP, GFAP, and S100. GFP expression was predominantly in enteric glia in myenteric ganglia (Supplementary Figure 2). Though rare, an occasional cell with neuronal morphology was detected (not pictured). Overall, the number of transduced glial cells was low (<5%). Further optimization is needed to increase glial targeting, however, these data show that AAV9 mediated transgene expression in the ENS can be biased toward enteric glia by changing the viral genome.

**Figure 3:**
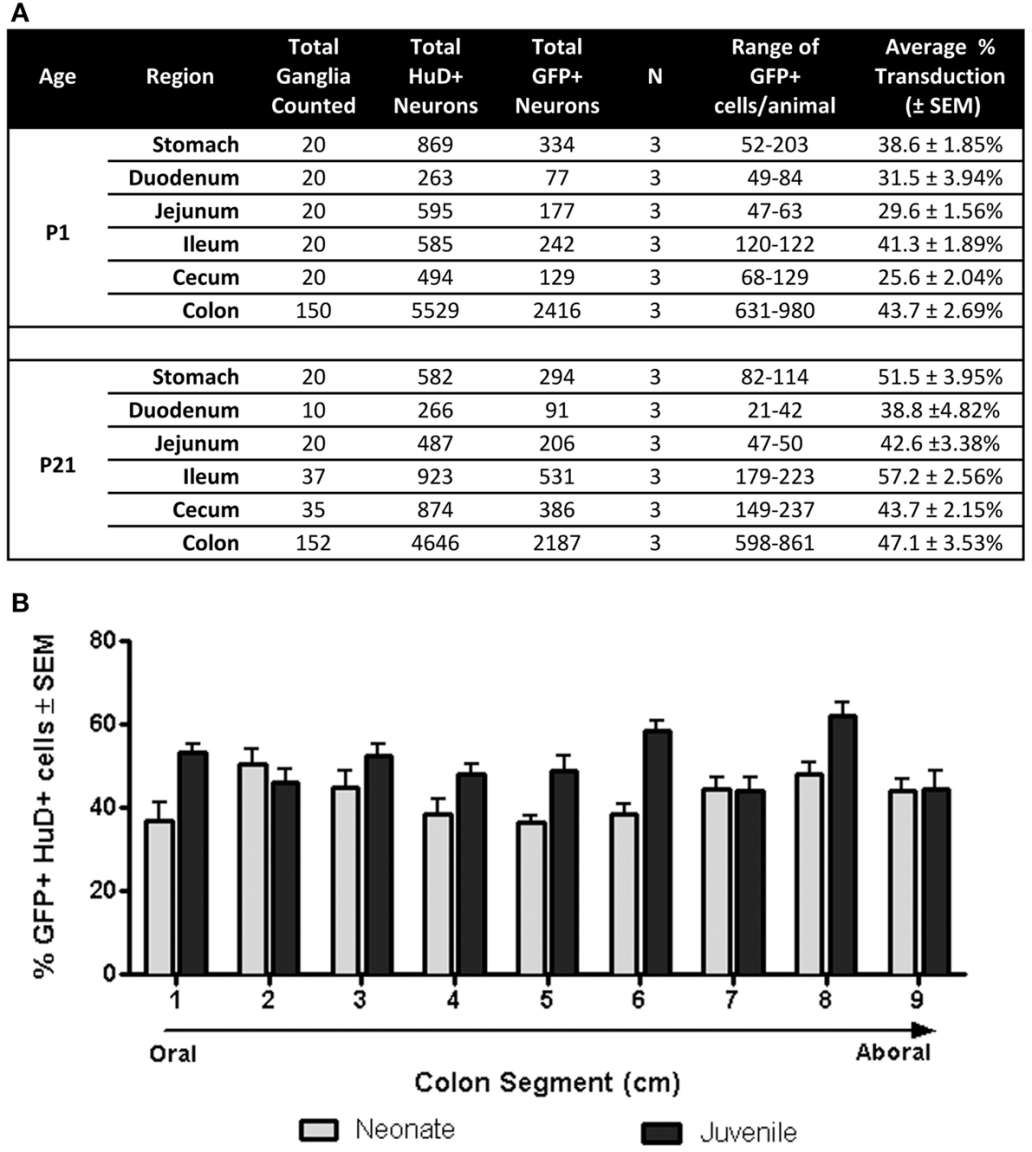
**Quantification of GFP expressing myenteric neurons. (A)** Total transduced and untransduced neuron counts in each gastrointestinal region in mice injected either at P1 (neonates, *n* = 3) or P21 (juveniles, *n* = 3). Transduction of HuD positive neurons ranged from ~25 to ~43% in neonatally injected mice. Transduction ranged from ~42 to 57% in juvenile injected mice. **(B)** Quantification of GFP myenteric neurons across the oral to aboral length of the colon revealed no transduction differences between proximal and distal colon segments in neonatal and juvenile injected mice.

Next we quantified GFP expressing myenteric neurons in mice that received scAAV9-CB-GFP injections as neonates or juvenile mice (*n* = 3 each group). In neonatal mice, average neuronal transduction ranged from 25 to 43% depending on GI region examined. GFP expression was most abundant in neurons of the ileum and colon, reaching 41.3 ± 2% and 43.7 ± 3%, respectively. Transduction ranged from 38 to 57% in juvenile injected mice. GFP expression was highest in the colon (47.1 ± 4%), stomach (51.5 ± 4%), and ileum (57.2 ± 3%). The difference in transduction efficiencies likely reflects differences in the doses administered (neonates = 6.7 × 10^10^ vg/g body weight and juveniles = 1.4 × 10^11^ vg/g body weight). These results are summarized in Figure 3A and demonstrate that efficient transduction of myenteric neurons can be achieved regardless of age of administration.

### AAV9 mediated GFP expression along the oral-aboral axis of the colon

Regions of the colon from oral to distal end (e.g., ascending, transverse, descending, sigmoid and rectum) are associated with anatomical differences in the size of ganglia and neuronal density (Hasler et al., 1990; Sibaev et al., 2003), as well as distinct functions and motor patterns of activity. To examine if myenteric plexus transduction was biased along the oral to aboral axis, GFP expression was analyzed in 1 cm colon segments (labeled 1–9; oral to aboral). Segments from P1 treated colons (*n* = 3) had GFP expression in 37–51% of myenteric neurons with no apparent gradient of GFP expression (Figure 3B). The highest regions of scAAV9 transduction were in segments 2 (50.8 ± 3%) and 8 (48.2 ± 3%), while the lowest were in segments 1 (36.9 ± 4%) and 5 (36.7 ± 2%). In colons of P21 injected animals (*n* = 3), GFP transduction peaked in segment 8 (62.1 ± 3%) followed closely by segments 6, 1, and 3 (58.4 ± 2, 53.3 ± 2, and 52.5 ± 3%, respectively). Segments 7 and 9 had the lowest percentage of GFP positive myenteric neurons (44.3 ± 3 and 44.5 ± 4%, respectively). A One-Way repeated measures ANOVA revealed no significant differences in GFP positive neurons between segments within groups (*p* > 0.05). Comparisons between ages were not considered.

### Chemical coding of AAV9 transduced neurons in the myenteric plexus

**Figure 4:**
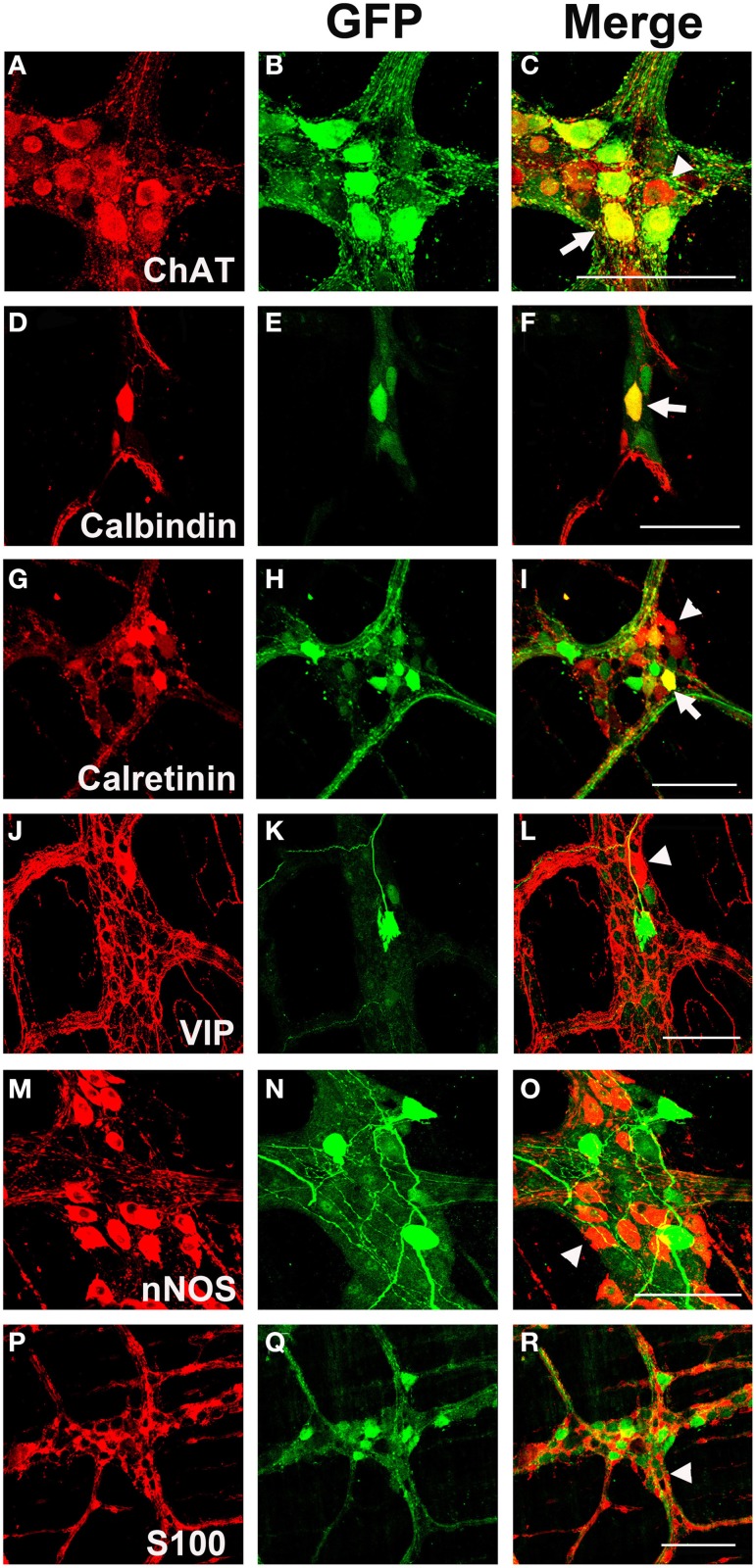
**Chemical coding of transduced myenteric cells in the colon after systemic scAAV9-CB-GFP injection**. GFP expression **(B,E,H,K,N,Q)** was detected in choline acetyltransferase (ChAT, **A,C**) positive, calbindin positive **(D,F)**, and calretinin positive **(G,I)** myenteric neurons and some intraganglioninc fibers. ChAT staining is indicative of excitatory motor neurons and ascending and descending projecting interneurons. Calbindin and calretinin calcium binding proteins are indicative of excitatory motor neurons and some classes of interneurons. Transduction was rare or completely absent in vasoactive intestinal peptide (VIP, **J,L**) positive or neuronal nitric oxide synthase (nNOS, **M,O**) positive cells. VIP and nNOS staining is associated with inhibitory motor neuron and descending interneurons. GFP did not co-localize with S100 positive glial cells **(P,R)**. Arrows indicate co-expression while arrowheads indicate no GFP expression. Scale bars are 100 μm.

Myenteric neurons can broadly be classified into motor neurons, interneurons and sensory neurons (Furness et al., 2003). These categories can be further delineated based on the direction of axonal projections and chemical markers present in cells, referred to as chemical coding (Costa et al., 1986; Lomax and Furness, 2000). To determine if specific neuronal types were preferentially transduced by scAAV9, LMMP preparations from the colon were triple labeled for GFP, HuD, and either calretinin, calbindin, nNOS, VIP, or ChAT. Neuronal subtypes expressing ChAT include excitatory motor neurons projecting to circular and longitudinal muscles, ascending and descending interneurons, and some sensory neurons (Harrington et al., 2010). VIP and nNOS labeling occurs in inhibitory motor neurons projecting to circular and longitudinal muscles (Furness, 2000). Calretinin expression occurs predominantly in ascending interneuron populations and less frequently in excitatory motor neurons (Bergner et al., 2014). Calbindin expression is commonly found in descending interneurons and sensory neurons (Costa et al., 1986; Furness, 2000). scAAV9 mediated GFP expression was common in neuronal cell bodies labeled with ChAT, calretinin, and calbindin when intravenously injected into P1 or P21 mice (Figures 4A–I). ChAT or calretinin labeled fiber tracts were also GFP positive. In contrast, GFP expression within VIP or nNOS positive neurons or interganglionic fibers was rare (Figures 4J–O). GFP expression was not detected in S100 positive glial cells (Figures 4P–R). Together, these data suggest preferential scAAV9-CB-GFP transduction of neurons involved in excitatory signaling.

### Transduction of the myenteric plexus following intravenous injection of scAAV1, scAAV5, scAAV6, and scAAV8 in neonatal mice

**Figure 5:**
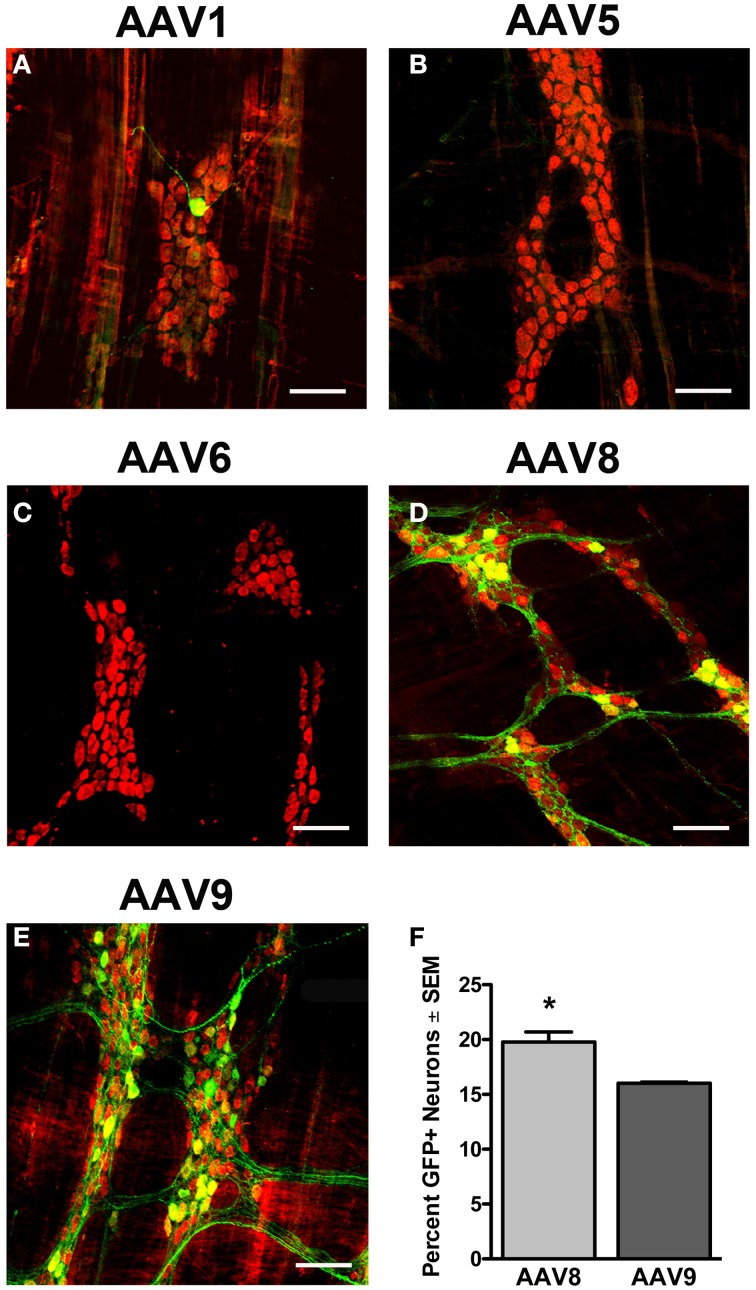
**Transduction efficiency in the myenteric plexus is dependent on AAV serotype**. Neonatal mice were intravenously injected with scAAV1 **(A)**, scAAV5 **(B)**, scAAV6 **(C)**, scAAV8 **(D)**, or scAAV9-CB-GFP **(E)** (final dose 5 × 10^10^ vg/ml). Green fluorescent protein (GFP, green) immunofluorescence in the myenteric plexus of the colon reveled absent to minimal GFP expression in neurons (HuD, red) following scAAV1, scAAV5, and scAAV6 intravenous injection. Robust GFP expression was detected in neurons in scAAV8 **(D)** and scAAV9 **(E)** injected mice. Total enumeration counts of neurons in the myenteric plexus of the colon revealed that 19.7 ± 0.9% or 16.0 ± 0.2% of neurons were transduced in scAAV8 and scAAV9 injected mice, respectively (**F**, ^*^p < 0.001). Scale bars are 100 μm.

**Figure 6:**
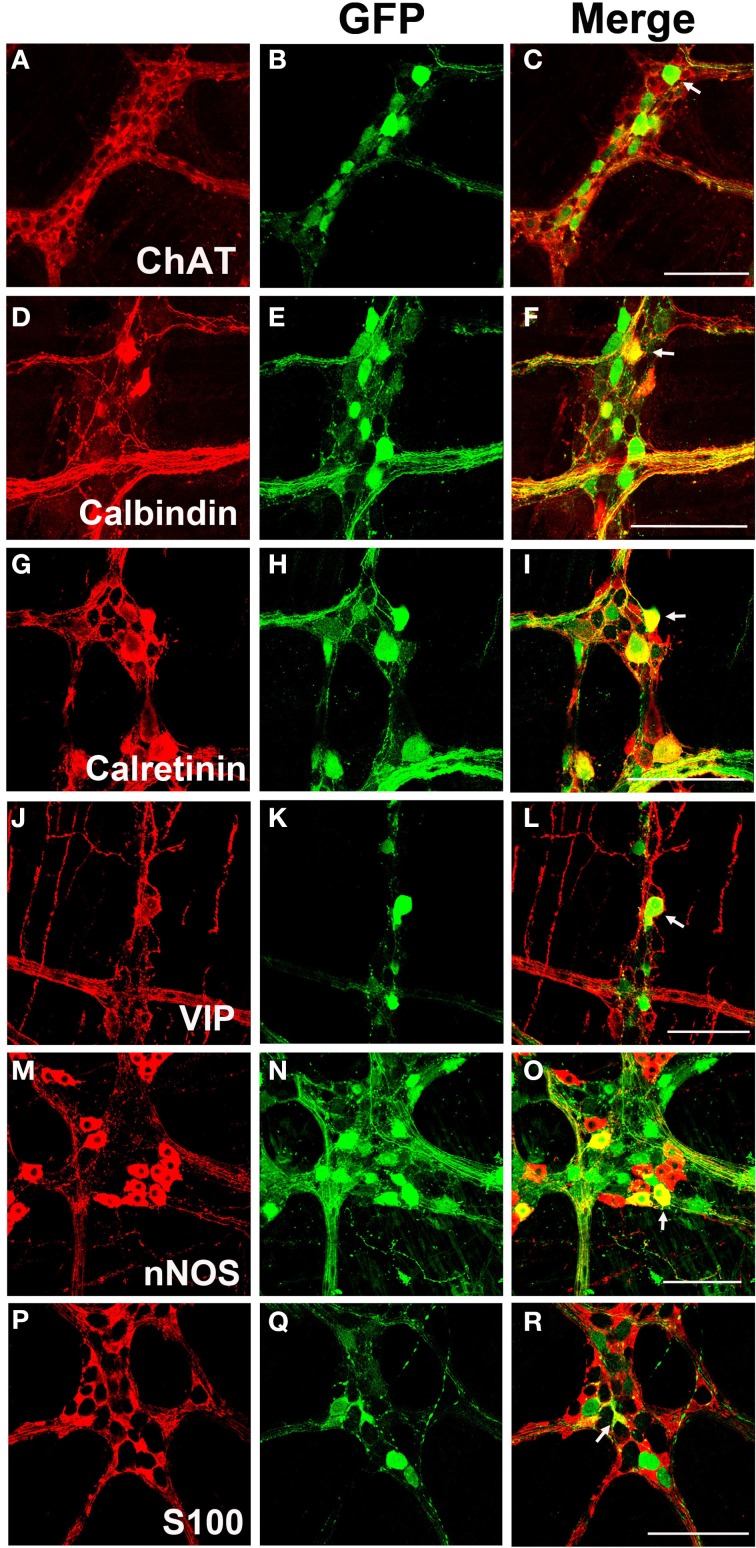
**Chemical coding of transduced myenteric cells in the colon after systemic scAAV8 injection**. Similarly to scAAV9, systemic injection of scAAV8-CB-GFP produced GFP expression **(B,E,H,K,N,Q)** was detected in ChAT **(A–C)**, calbindin **(D–F)**, and calretinin **(G–I)** positive excitatory neurons, interneurons and intraganglionic fibers. Additionally, GFP expression was also detected in VIP **(J–L)** and nNOS **(M–O)** positive inhibitory neurons, interneurons, and intraganglionic fibers. GFP expression was occasionally detected in S100 positive enteric glia **(P–R)**. White arrows in merged images indicate transduced cells. Scale bars are 100 μm.

To determine if a serotype capable of vascular escape was required to transduce the myenteric plexus, we tested scAAV serotypes that efficiently traverse (AAV6, 8, and 9) or are inhibited (AAV1 and 5) by endothelial cell barriers (Supplementary Figure 3). We intravenously injected neonate mice with 5 × 10^10^ vg of either scAAV1, scAAV5, scAAV6, scAAV8, or scAAV9 that encoded identical expression cassettes (scAAV-CB-GFP). Animals were euthanized 30–60 days post-injection and the myenteric plexus was immunolabeled for GFP, HuD, and S100. Immunolabeling revealed limited to no transduction of myenteric neurons or glial cells following AAV1, 5, and 6 injection (Figures 5A–C). scAAV8 injection produced robust, widespread transduction in the myenteric plexus in all regions of the GI tract. AAV8 mediated GFP expression was observed in myenteric neurons, fiber tracts, and some intraganglionic glial cells (Figures 5D, 6). Neuronal transduction in the colons of scAAV8 and scAAV9 treated mice was quantified. A total of 3029 HuD positive and 599 GFP positive neurons from 91 ganglia were counted in scAAV8 injected mice. A total of 3366 HuD positive and 539 GFP positive neurons in 90 ganglia were counted from scAAV9 injected mice (Figure 5F). A Student's *t*-test revealed significantly higher percentage of myenteric neuron transduction in scAAV8 injected mice (average 20.6 ± 1%) compared to scAAV9 injected mice (15.8 ± 1%) at the 5 × 10^10^ vg dose [*t*_(179)_ = 4.11, *p* < 0.001]. These data show that AAV must be able to escape vasculature following systemic injection to efficiently transduce myenteric plexus.

### AAV8 mediated transgene expression in myenteric neuron subtypes and enteric glia

LMMP tissues from scAAV8-CB-GFP were fluorescently immunolabeled for GFP, HuD, and VIP, nNOS, calretinin, calbindin, and ChAT. Similarly to scAAV9, scAAV8 mediated GFP expression occurred in ChAT, calretinin and calbindin positive myenteric neurons (Figures 6A–I). Additionally, robust GFP expression was detected in calretinin and calbindin positive interganglionic fibers. Contrary to scAAV9, GFP expression was also detected in VIP and nNOS expressing inhibitory motor neurons with limited co-expression in fibers (Figures 6J–O). Furthermore, GFP expression was sparingly detected in intraganglionic S100 positive EGCs (Figures 6P–R).

## Discussion

Prior to this work, AAV transduction of the GI tract and the ENS was not well characterized (Fu et al., 2011; Rahim et al., 2011; Mattar et al., 2013; Schuster et al., 2014). Understanding of AAV transduction in the ENS is essential for therapeutic development in neurological disease, FGIMD, GI neuromuscular disease, FGID's, IBS, diarrheal diseases and IBD. Here we examined transduction of the myenteric plexus following systemic delivery of scAAV9 and additional serotypes scAAV1, 5, 6, and 8. scAAV9 transduced myenteric neurons along the entire GI tract when systemically administered to neonate or juvenile mice. Dosing was not controlled for body weight across ages therefore comparisons of transduction efficiency dependent on age cannot be made. Despite that, scAAV9 transduction was robust when administered at either age, achieving transduction of 25–57% of myenteric neurons across various regions of the GI tract. Structural variations along the colon did not affect transduction in the myenteric plexus. Furthermore, we report that AAV serotypes that efficiently traverse the BBB in the CNS are necessary but may not be sufficient for myenteric neuron transduction (Figure 5 and Supplementary Figure 3).

The extensive overlap of the more than 40 neurotransmitters identified in the ENS limits interpretation of the cells targeted by AAV (Furness, 2012). However, we examined colocalization between GFP and known ENS markers including ChAT, VIP, nNOS, calbindin, calretinin, and S100. scAAV9 transgene expression was more common in neurons involved in excitatory neurotransmission (ChAT), sensation (calbindin), and ascending signaling (calretinin). In general, viral tropism is dictated by the capsid and expression cassette and is somewhat malleable. For example, substitution of a GFAP promoter for the ubiquitous chicken β-actin (CB) promoter in scAAV9 was effective at targeting enteric glia (Supplementary Figure 2). Because the CB promoter produces robust expression in CNS astrocytes but not in enteric glia, this finding further emphasizes that enteric glia are similar yet distinct from CNS astrocytes (Gulbransen and Sharkey, 2012; McClain et al., 2014). Importantly, the term “enteric glia” encompasses multiple subtypes of glial cells with divergent morphologies and localities within the gut wall (Gulbransen and Sharkey, 2012). Cellular properties of enteric glia subtypes, their interactions with ENS vasculature, and how those factors compare to CNS astrocytes is not well understood. Functional and physiological disparities from CNS astrocytes may explain contrasting efficiencies in glial cell transduction within the ENS and CNS following juvenile AAV9 systemic delivery. The emerging role of enteric glia in the tripartite synapse of the ENS as in CNS, in GI motility, and their likely contribution to GI motility disorders and gut inflammation make them an important target within the ENS and merits further investigation (Lomax et al., 2005; Ren et al., 2011; Gulbransen and Sharkey, 2012; McClain et al., 2014). Further variation in enteric cell tropism is provided by changing the AAV capsid.

Despite all of the tested serotypes having neuronal tropisms in the CNS, only scAAV8 had robust myenteric transduction similar to scAAV9. Interestingly, scAAV8 transduction included VIP and nNOS neurons and enteric glia that were rarely seen in scAAV9 tissue. This broader transduction profile seen with scAAV8 suggests that the AAV catalog may allow for powerful customization of ENS gene delivery. Differences in receptors for scAAV8 and scAAV9 likely contribute to the observed differences in myenteric transduction (Akache et al., 2006; Bell et al., 2011; Shen et al., 2011). In contrast to scAAV8 and 9, systemic injection of scAAV1, 5, and 6 produced few to no GFP positive cells. These data suggest that vascular escape is a requirement for AAV mediated myenteric neuron transduction and is in agreement with prior work showing that ENS vasculature is not fenestrated (Gershon and Bursztajn, 1978).

Systemic AAV gene delivery may be beneficial for multi-system neurological disease. Patients with CNS disorders like Alzheimer's, Parkinson's, autism spectrum disorders, spinal cord injury and neuromuscular diseases have reported GI complications which likely involve ENS pathology (Anderson et al., 2007; Buie et al., 2010; Wakabayashi et al., 2010; Natale et al., 2011; Mazurek et al., 2012). Many of these disorders are the subjects of gene therapy studies (Kaplitt et al., 2007; Foust et al., 2010; Muramatsu et al., 2010; Garg et al., 2013). Currently, the high doses of systemic AAV delivery required for efficient CNS transduction limits usage in adults. However, delivery into cerebrospinal fluid (CSF) or direct CNS injection in combination with low dose systemic delivery could simultaneously treat CNS and GI aspects of neurological disease (Bevan et al., 2011; Samaranch et al., 2012). Indeed, Parkinson's and spinal cord injury patients list GI dysfunction as severely impacting quality of life (Sakakibara et al., 2001; Anderson, 2004). Therefore, ideal therapies for these disorders will also remedy GI dysfunction.

The current findings are also important for studies of ENS biology. Transgenic approaches to study the ENS can be hampered by the degree of overlap between ENS and CNS cells. For example, most cre drivers designed for nervous tissue express in both the CNS and ENS which complicates experimental design. Therefore, development of AAV vectors with restricted expression would be useful in basic studies of ENS biology. Such a vector could be used to deliver a cDNA or RNAi construct to manipulate gene expression exclusively within the ENS. Additionally, an AAV can exogenously express cre-recombinase and add a temporal component to ENS studies. Alternatively, AAV vectors can be used in knockout animals to maintain tissue specific expression of a targeted gene. Gene expression can be restricted to the CNS by intracerebroventricular (ICV) injection. ICV injection in mice results in minimal gene expression in peripheral tissues. Following ICV injection, resulting animals would be chimeric for the knocked out gene; with CNS expression maintained and ENS expression depleted. Finally, the current data can likely be translated to other species, such as guinea pig or non-human primates, where genetic tools are limited. The pattern of scAAV9 transgene expression has been consistent across mice, rats, cats and non-human primates. This would allow fluorescent labeling or genetic manipulation of the ENS in novel species.

In summary, results from the current study demonstrate that systemic AAV delivery can efficiently target the myenteric plexus in the entire gastrointestinal tract in mice. These data should lay the foundation for therapies directed at GI motility disorders, neurological disease and inflammatory diseases affecting enteric glia. Future efforts will refine the ENS neuronal and glial cell subtypes targeted and evaluate transduction within the submucosal plexus.

## Author contributions

Kevin D. Foust, Fedias L. Christofi, and Sara E. Gombash conceived and designed the studies. Sara E. Gombash, Christopher J. Cowley, and Julie A. Fitzgerald performed the studies. Christian Mueller and Jodie C. E. Hall helped in vector development and production. Sara E. Gombash and Kevin D. Foust wrote the manuscript. All authors contributed to the editing and preparation of the manuscript.

### Conflict of interest statement

The authors declare that the research was conducted in the absence of any commercial or financial relationships that could be construed as a potential conflict of interest.

## Supplementary material

Supplementary Figure 1 **GFP expression is confined to myenteric neurons**. Systemic injection of scAAV9-CB-GFP resulted in transduction of myenteric neurons in both neonatal (P1, **A–E**) and juvenile (P21, **F–J**) injected mice. Co-expression in neurons but not enteric glia was confirmed by immunohistochemistry for GFP **(A,F)**, S100 identifying enteric glia **(B,G)**, and HuD identifying myenteric neurons **(D,I)**. Merged images show GFP expression in neurons only **(C,H,E,J)**. Arrows show GFP expression in neurons that does no coincide with S100 glia staining. Scale bars are 100 or 50 μm in insets.

Supplementary Figure 2 **Altered myenteric cell type transduction following change in AAV promoter**. GFP expression **(A)** was detected exclusively in S100 positive **(B,C)** myenteric glial cells following intravenous administration of ssAAV9-GFAP-GFP.

Supplementary Figure 3 **AAV Transduction in the Brain and Spinal Cord following intravenous injection**. GFP immunofluorescence was detected in neurons (NeuN, cyan) and astrocytes [glial fibrillary acidic protein (GFAP), red] in the brains and spinal cords of scAAV1 **(A,F)**, scAAV6 **(C,H)**, scAAV8 **(D,I)**, and scAAV9 **(E,J)** CB-GFP intravenously injected mice. No CNS transduction occurred in scAAV5 **(B,G)** injected animals. Arrowheads indicate transduced neurons (co-labeling with NeuN) and arrows indicate transduced astrocytes (co-labeled with GFAP). Scale bars are 100 μm.
