# Supplementary figures and images for: Intravenous AAV9 efficiently transduces myenteric neurons in neonate and juvenile mice
Source: Front Mol Neurosci. 2014 Oct 15;7:81. doi: 10.3389/fnmol.2014.00081 (PMC4197761; doi:10.3389/fnmol.2014.00081)

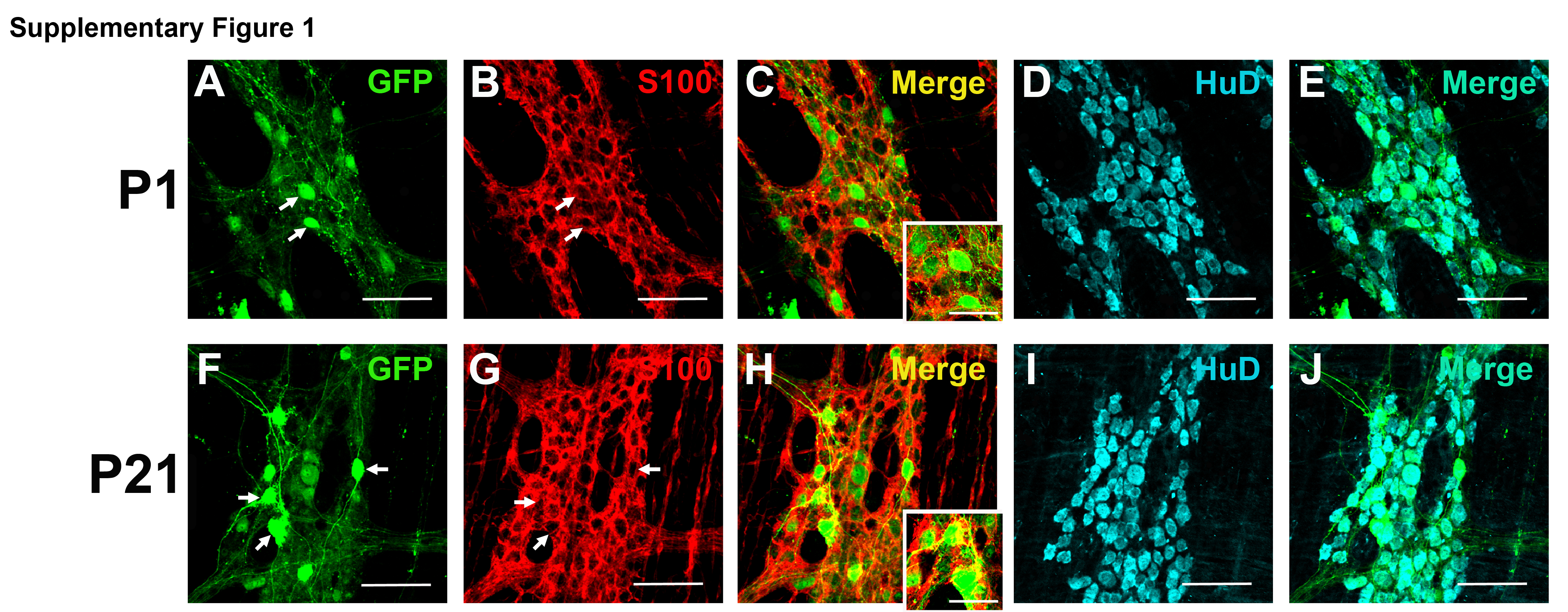

Supplement: Supplementary Figure 1 — GFP expression is confined to myenteric neurons. Systemic injection of scAAV9-CB-GFP resulted in transduction of myenteric neurons in both neonatal (P1, A–E) and juvenile (P21, F–J) injected mice. Co-expression in neurons but not enteric glia was confirmed by immunohistochemistry for GFP (A,F), S100 identifying enteric glia (B,G), and HuD identifying myenteric neurons (D,I). Merged images show GFP expression in neurons only (C,H,E,J). Arrows show GFP expression in neurons that does no coincide with S100 glia staining. Scale bars are 100 or 50 μm in insets. [file Image1.JPEG]

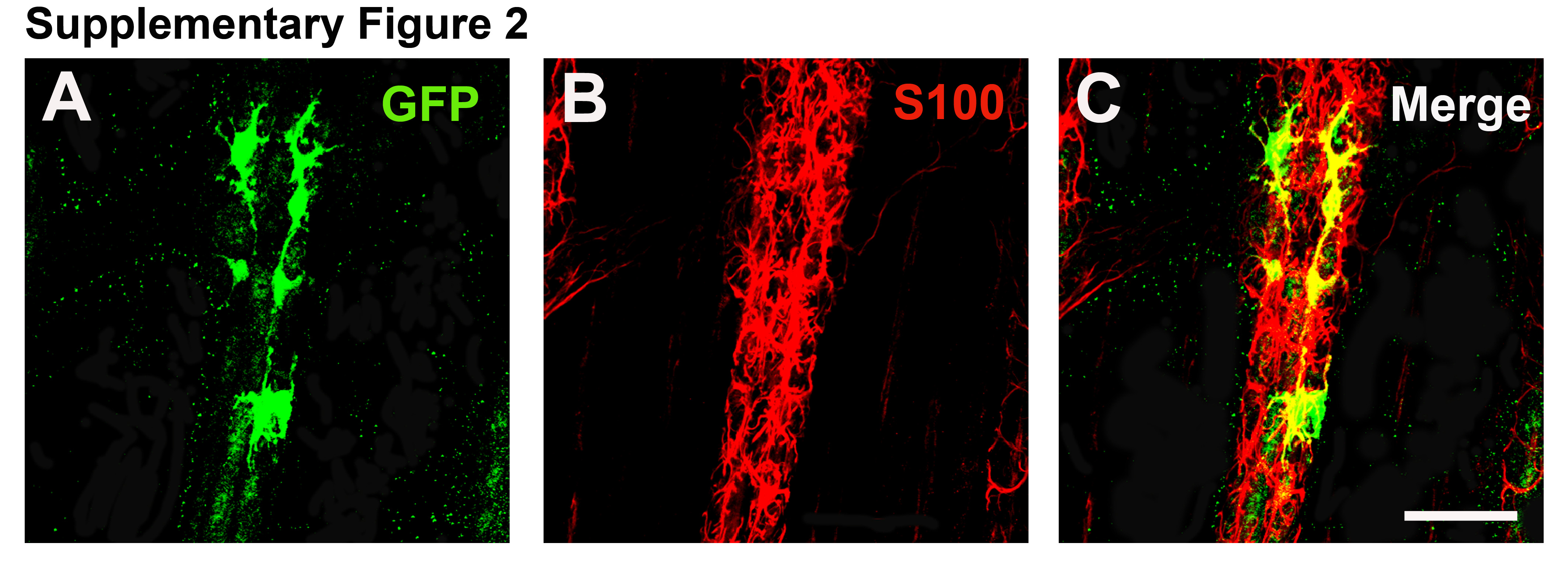

Supplement: Supplementary Figure 2 — Altered myenteric cell type transduction following change in AAV promoter. GFP expression (A) was detected exclusively in S100 positive (B,C) myenteric glial cells following intravenous administration of ssAAV9-GFAP-GFP. [file Image2.JPEG]

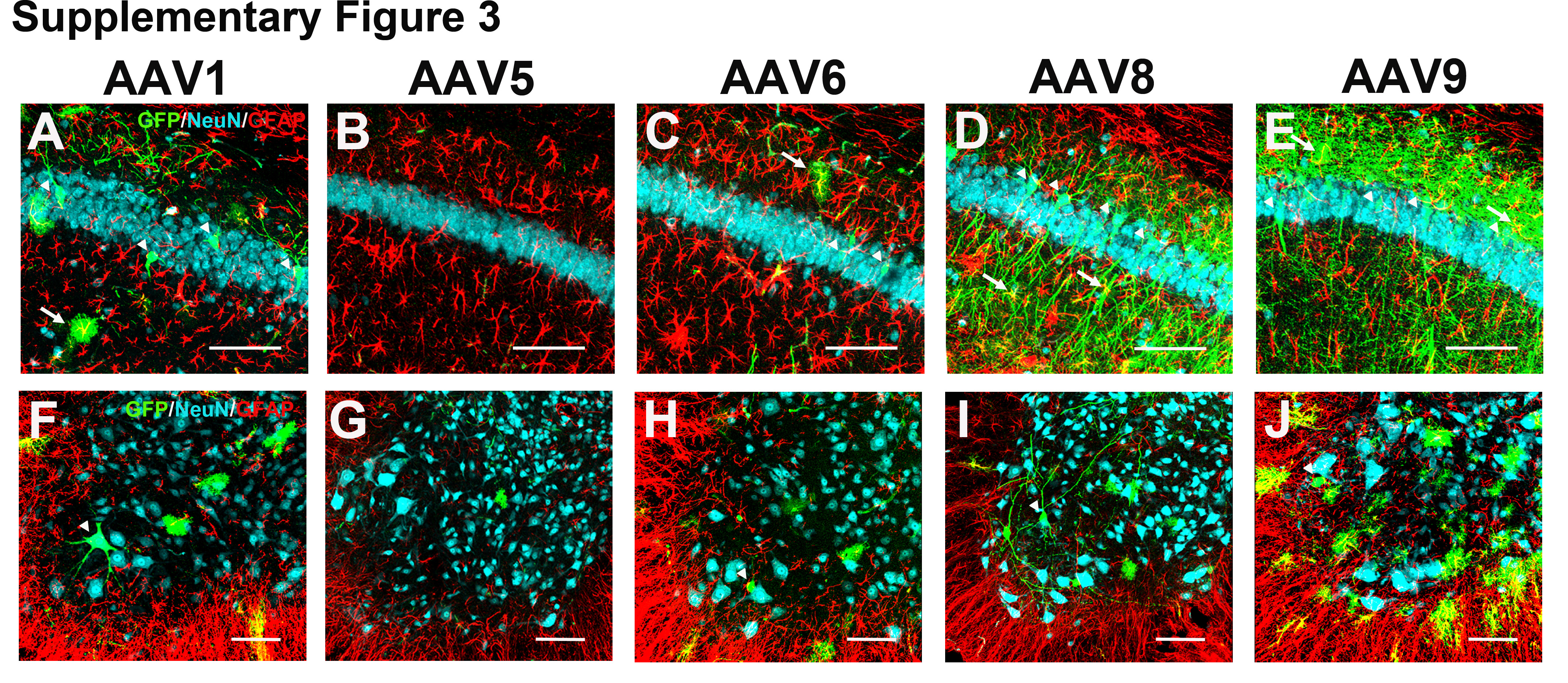

Supplement: Supplementary Figure 3 — AAV Transduction in the Brain and Spinal Cord following intravenous injection. GFP immunofluorescence was detected in neurons (NeuN, cyan) and astrocytes [glial fibrillary acidic protein (GFAP), red] in the brains and spinal cords of scAAV1 (A,F), scAAV6 (C,H), scAAV8 (D,I), and scAAV9 (E,J) CB-GFP intravenously injected mice. No CNS transduction occurred in scAAV5 (B,G) injected animals. Arrowheads indicate transduced neurons (co-labeling with NeuN) and arrows indicate transduced astrocytes (co-labeled with GFAP). Scale bars are 100 μm. [file Image3.JPEG]
